# One-Health Challenge in H9N2 Avian Influenza: Novel Human-Avian Reassortment Virus in Guangdong Province, China

**DOI:** 10.1155/2024/9913934

**Published:** 2024-02-13

**Authors:** Qiucheng Yao, Jing Liu, Huizhen Liu, Yan Zhou, Miaotong Huo, Yuanguo Li, Yuwei Gao, Ye Ge

**Affiliations:** ^1^College of Coastal Agricultural Sciences, Guangdong Ocean University, Zhanjiang, China; ^2^Military Veterinary Research Institute of Academy of Military Medical Sciences, Changchun, China

## Abstract

China is one of the highest producers of poultry meat output in the world, with a large scale of chicken rearing. Statistically analyzed H9N2-subtype avian influenza viruses (AIVs) have become the dominant subtype in China's live poultry market, with the highest detection rate. Although H9N2 AIV is of low pathogenicity and tends not to cause serious disease and high mortality in poultry, it poses a great challenge to the domestic poultry farming industry by causing a decrease in appetite, a decline in egg production, and deaths caused by mixed infections with another pathogenic microorganism. Moreover, novel influenza viruses (H7N9 and H3N8) infecting humans have emerged in China, and the H9N2 AIV provides all or part of the internal genes to the new recombinant viruses, posing a potential threat to public health and safety and human health. In this research, six H9N2 AIVs were isolated from feces or oropharyngeal swabs collected from live poultry markets and duck farms in Zhanjiang. After epidemiological investigations, phylogenetic analyses, and molecular characterization, we found that the ZJ81 strain was a chicken–human–mink recombinant virus, the ML3 strain was a chicken-human recombinant virus, and all six virus strains of the virus had a bias for the human receptor-binding site and a mutation that could cause an increase in virulence in mice. Therefore, surveillance and control of H9N2 AIV should be strengthened to provide data support for cross-species transmission of H9N2 AIV.

## 1. Introduction

Avian influenza (AI) was first reported in Italy in 1878, with outbreaks and high mortality in poultry, an event called “avian plague.” However, it was not recognized as an influenza A virus until 1955, and since then, avian influenza viruses (AIVs) have been detected throughout the country [1].

Influenza A viruses are single-stranded, negative-stranded, segmented RNA viruses consisting of 8 gene fragments encoding 12 proteins [2]. Hemagglutinin (HA), as a surface protein of viruses, is one of the determinants of viral virulence, as it can recognize host receptors and induce the fusion of viral vesicles and cell membranes [3]. Neuraminidase (NA) and some studies have indicated that a shorter length of the NA stem enhances the ability of the virus to bind to the receptor and enhance the virulence of the virus in mice [4, 5]. Influenza viruses are classified into subtypes based on their surface antigens, HA and NA. To date, 18 HA and 11 NA subtypes have been identified from birds and bats [2]. Nuclear proteins (NPs) are involved in the transcription and replication of viral genomes [6]. The polymerase complex proteins are composed of PA, PB1, and PB2 together. The PB1 protein is the core of the influenza virus RNA polymerase complex. More and more studies are located on PA protein' host adaptation. There are many amino acid sites as a key on the PB2 protein, such as E627 K and D701 N, which affect the pathogenicity, host adaptability, and transmission capacity of the virus [5]. NS1 plays a key role in preventing the host immune response by inhibiting Type I interferons and influences viral replication, propagation, and virulence. NEP plays a crucial role in the export of the viral genome from the nucleus, regulating viral transcription and replication [7]. The M gene encodes two proteins, M1 and M2, which, together with the vRNP complex, carry out nuclear and cytoplasmic transport and play a key role in the packaging, morphology, and transcription of viral particles. Another, the M2 protein acts as an ion channel, the depolymerization of protein complexes, and the release of ribonucleoproteins. Some researchers have demonstrated that M2 protein can also affect virus replication by influencing cellular autophagy [8]. The PB1-F2 encode in PB1 played an important role in enhancing the virulence of H5N1 viruses. PA-X, encoded by the PA gene, plays a role in viral replication, viral induction of apoptosis, and virulence [9].

In 1966, the first strain of H9N2-subtype AIV was detected from turkeys in Wisconsin, USA, and has since become globally widespread [10]. The H9N2 virus was classified into two main branches according to epidemiologic studies and phylogenetic analyses: the North American lineage and the Eurasian lineage. The Eurasian lineage is further divided into three branches: A/chicken/Beijing/1/94-like (BJ/94-like), A/quail/HongKong/G1/97-like (G1-like), and A/duck/HongKong/Y439/97 (Y439-like) [11]. The BJ/94-like lineage in mainland China is predominantly found in poultry. H9N2-subtype AIV has a wide host range. It is not only common in poultry and wild birds but also cross-infects mammals, especially humans [12]. In 1998, the first human case of H9N2 AIV was observed in China. Since then, many cases have emerged. Most importantly, H9N2 AIVs could become a donor for other influenza viruses. That is, H9N2 AIVs provide gene fragments and recombines with other subtypes of AIVs. Notably, human-infecting recombinant AIVs have acquired all or part of the internal genes of H9N2 AIVs; examples include H5N1 in 1997, H7N9 in 2013, and others (H10N8, H5N6, H6N1 and H7N4) [13–19]. According to the latest reports, the six internal genes of the H3N8 virus, which causes human infection, are all derived from the H9N2 AIV of chicken origin [20].

H9N2-subtype AIV first appeared in Guangdong, China, in 1992. This virus spread rapidly throughout China and caused a large-scale outbreak of H9N2 in 1998, which spread from farms throughout the country in a short time [21, 22]. In the same year, the first human was infected with H9N2 AIV in China. H9N2 is still spreading in poultry farms today and has become one of the major influenza viruses affecting animals on farms. According to research studies, H9N2 AIV has been replaced H5N6 and H7N9 as the major subtypes in China [23]. At present, there is a serious influenza epidemic in European countries, such as AI A subtype H5. The authorities concerned have initiated animal culling and outbreak investigations, and tens of thousands of poultry have been culled. We have therefore carried out a series of work to assess the health conditions of poultry and wild birds.

In this research, we mainly sampled the live poultry trading market and farms in Zhanjiang City, Guangdong Province, from 2019 to 2021. The whole genomes of these H9N2 AIVs were sequenced and analyzed by epidemiological investigations, phylogenetic analyses, and molecular characterization. Our study provides strong data to support the prevention of cross-species spread of H9 = N2 AIV in domestic poultry.

## 2. Materials and Methods

### 2.1. Sample Collection and Sequence Information

We chose to randomly collect fresh feces, oropharyngeal and cloacal swabs from chickens, ducks, and geese at live poultry markets in the east, south, west, and north of the Leizhou Peninsula and assessed the health status of several representative farms. A total of 227 samples were collected. The oropharyngeal and/or cloacal swabs were placed into centrifuge tubes containing penicillin, streptomycin, and glycerol of phosphate-buffered saline. The sample tubes were stored at low temperatures, transported immediately to the lab, and frozen in a refrigerator at −80°C.

5742 HA sequences of the H9N2 AIVs from 2017 to July 2023 were downloaded from the GISAID database (GISAID gisaid.org) and the NCBI database (Influenza Virus Database, NCBI (nih.gov)). The repeat sequences that had the same ID in the NCBI and GISAID databases were removed. The retained sequences were subjected to host (wild bird, poultry, human, and mammal) counts, isolation area counts, and isolation time counts, and correlation charts were created to reflect the prevalence of the H9N2 subtype of AIV.

### 2.2. Influenza Virus Isolation and Identification

SPF chicken embryos of 9–11 days of age were selected, and the processed samples were inoculated into the allantoic cavity, 0.2 ml/embryo [24]. After inoculation, the embryos were wax-sealed and incubated in an incubator at 37°C for 72 hr. The embryos were photographed after 24 and 48 hr, and the dead embryos were removed and stored in a refrigerator at 4°C. After 72 hr, the chicken embryos were placed in a 4°C refrigerator overnight. Allantoic fluid was aseptically harvested from chicken embryos, and 1% of chicken red blood cells were configured for hemagglutination activity assay, and hemagglutination-active viral fluids were preserved. RNA extraction and viral reverse transcription of the allantoic fluid, and the above steps according to the instructions of the reagent. The primers used to amplify the eight genes' fragments of AIVs by RT-PCR will be provided upon reasonable request. The whole genome sequence of AIV was determined by second-generation sequencing. The sequencing data were merged and assembled for processing.

### 2.3. Model Selection and Evolutionary Dynamics

Sequence splicing of the whole genome was performed by SeqMan in DNASTAR 11.0 software; the sequences were analyzed by BLAST comparison, and the top 100 sequences with the highest nucleotide identity and the representative sequences were downloaded as reference sequences. The six inner genes segment sequences with the full length of the coding region were retained to generate a genetic evolutionary tree using the maximum likelihood (ML) method and 1,000 bootstrap replications.

For the surface gene, multiple sequence comparisons were performed using MAFFT, including the H9N2 sequence. The duplicate and incomplete information sequences were deleted. The optimal nucleotide substitution model was confirmed by PhyloSuite (v1.2.2) software. TempEst (v.1.5.3) software was used to evaluate the data for the presence or absence of a time signal for molecular clock analysis [25]. The time to the most common ancestor was evaluated using the BEAST package (v1.8.4) with the GTR-F-R4 nucleotide substitution model for HA sequences and the GTR-F-R3 nucleotide substitution model for NA sequences and by choosing the appropriate clock model. Markov chain Monte Carlo runs 50 million states with sampling every 5,000 steps, ensuring an effective sample size of more than 200 [26, 27]. Each tree ran three times independently and was combined by Log Combiner. After a burn-in of 10%, the final tree was summarized using TreeAnnotator. The tree was summed using TreeAnnotator and embellished in FigTree (v1.4.4) software [27].

### 2.4. Analysis of Key Amino Acid Mutation Sites of H9N2 Viruses

Sequence homology analysis was performed using the DNASTAR 11.0 software MegAlign package, and noncoding regions of the sequences were deleted. The H9N2 AIV protein sequence obtained in this experiment was translated for cleavage site and important amino acid site analysis.

### 2.5. Selection Analysis

Sequences downloaded from the NCBI were processed with MEGA 11. 0, coding regions were retained, and their non-synonymous substitutions (*dN*), synonymous substitutions (*dS*), and *dN*/*dS* ratios were computed using the software Launch DnaSP6.

### 2.6. Structural Simulation of the H9N2 AIV HA Protein

The protein structure was predicted for A/Duck/Guangdong/ML3/2019 AIV. The 3D structure of the HA protein of AIV was constructed by using the Alphafold2 online site [28], and the best model was selected. The molecular model of HA protein was labeled with amino acid mutational sites in different colors. The model was annotated on Pymol version 2.6.0.

## 3. Results

### 3.1. Isolation, Identification, and Epidemiology of H9N2 AIVs

Six virus strains of AIVs subtype H9N2 were isolated from 227 samples for a positivity rate of 2.6% in this research. These six virus strains were named as follow: A/chicken/Guangdong/ZJ81/2021 (ZJ81); A/chicken/Guangdong/ZJ95/2021 (ZJ95); A/duck/Guangdong/ML3/2019 (ML3); A/ahicken/Guangdong/DF4/2019 (DF4); A/chicken/Guangdong/ZJ92/2021 (ZJ92); A/chicken/Guangdong/DF10/2019 (DF10). The full-length sequencing of each of the genes PB2 (2,280 nt), PB1 (2,274 nt), PA (2,151 nt), HA (1,683 nt), NP (1,497 nt), NA (1,401 nt), M (971 nt), and NS (838 nt) were obtained by second-generation sequencing.

**Figure 1:**
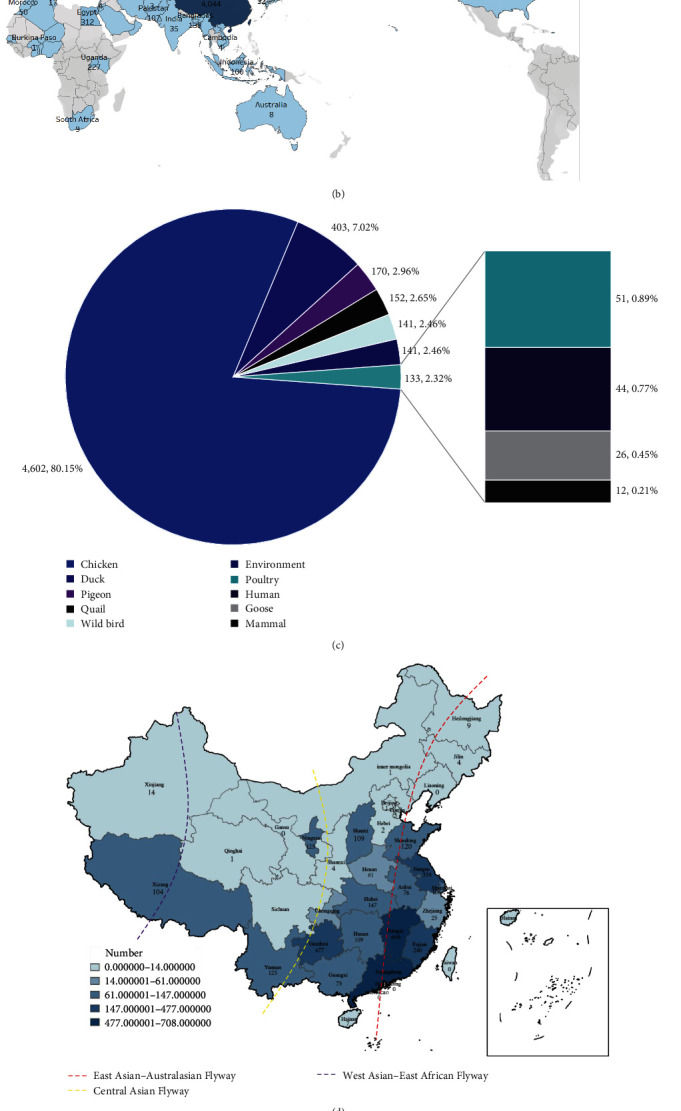
H9N2 AIV epidemiological survey performed from 2017 to July 2023 in worldwide. (a) Number of H9N2 AIV sequences downloaded from the NCBI and GISAID gene banks from 2017 to July 2023. (b) Global geographic location and population distribution of H9N2 viruses. (c) Distribution of H9N2 virus hosts. (d) Geographic and quantitative distributions of H9N2 viruses in China. The dotted lines indicate the three major migration routes through China. (e) Positive isolation rate of AIV in China, 2017–July 2023: Northeast China (Heilongjiang, Jilin, Liaoning), North China (Beijing, Tianjin, Hebei, Shanxi, Inner Mongolia), Northwest China (Shaanxi, Gansu, Qinghai, Ningxia, Xinjiang), Central China (Henan, Hubei, Hunan), Eastern China (Shandong, Jiangsu, Anhui, Shanghai, Zhejiang, Jiangxi, Fujian, Taiwan), Southern China (Guangdong, Guangxi, Hainan, Hong Kong, Macau) and Southwest China (Chongqing, Guizhou, Yunnan, Tibet, Sichuan).

Statistical analysis was done for different H9N2-subtype AIVs worldwide from January 2017 to July 2023. The results showed that the highest number of H9N2 AIVs were isolated in 2018, 2,714 strains. The number of viruses isolated then plummeted in 2019, with a trend of decreasing numbers in subsequent years. To date, only five sequences have been identified for 2023 (Figure 1(a)). The most significant number of H9N2 strains were isolated in China (4,044 strains), accounting for 70.4% of the global isolates of H9N2 AIVs, followed by Egypt (312 strains), Vietnam (218 strains), Uganda (227 strains), Bangladesh (142 strains), Israel (109 strains), Pakistan (107 strains) and Indonesia (100 strains). Fewer strains have been isolated in other countries (Figure 1(b)).

H9N2-subtype AIV has a wide host range. In the past few years, this type of virus has been detected from a variety of wild birds, poultry, and mammals. According to the data, most virus strains were isolated from chickens (4,602 strains), ducks (403 strains), geese (26 strains), pigeons (170 strains), quail (152 strains), the environment (141 strains), wildfowl (141 strains), humans (44 strains), bats (2 strains), 12 strains mammals, including mink, civets, cats, badgers, swine, and masked palm civet, and a well-defined category of poultry (51 strains), with chickens being the most susceptible to H9N2 AIV and being the primary host (Figure 1(c)). China is the center of H9N2 AI, dividing 23 provinces into seven regions: the Northeastern region, including Heilongjiang, Jilin, and Liaoning; North China, including Beijing, Tianjin, Hebei, Shanxi, and Inner Mongolia; Northwest China, including Shaanxi, Gansu, Qinghai, Ningxia, and Xinjiang; Central China, including Henan, Hubei, and Hunan; Eastern China, including Shandong, Jiangsu, Anhui, Shanghai, Zhejiang, Jiangxi, Fujian, and Taiwan; Southern China, including Guangdong, Guangxi, Hainan, Hong Kong, and Macau; Southwest China, including Chongqing, Guizhou, Yunnan, Tibet, and Sichuan. These seven districts had AIV positivity rates of 0.35%, 3.11%, 3.86%, 8.94%, 43.53%, 21.11%, and 19.56%, respectively, for the period from 2017 to July 2023. It is worth noting that the AIV positivity rate was only 0.35% in the northeast, with higher rates in Eastern China, Southern China, and Southwest China, where AIV is predominantly endemic (Figures 1(d) and 1(e)).

### 3.2. Phylogenetic Analysis

**Figure 2:**
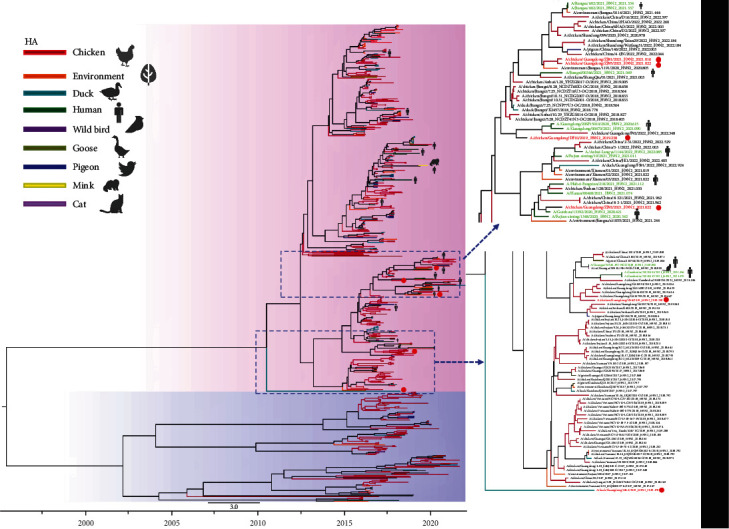
Phylogenetic tree of the HA genes of H9N2-subtype AIVs. Evolutionary trees with Bootstrap values >70% were generated by 1,000 bootstrap replicates using Bayesian inference. Viruses are labeled with different colors depending on the host (red: chicken, orange: environment, blue–green: duck, dark blue: pigeon, dark green: human, purple: wild bird, brown: goose, yellow: mink, light purple: cat). The red circles and font show the six H9N2 AIV strains isolated in this study, and the green font shows human influenza (H9N2). The pink background indicates the Eurasian branch and the blue background indicates the North American branch.

**Figure 3:**
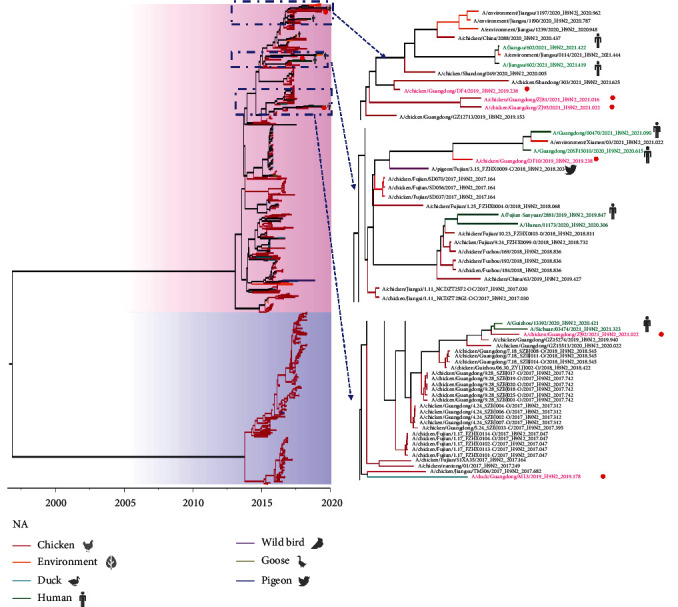
Phylogenetic tree of the NA genes of H9N2-subtype AIVs. Evolutionary trees with Bootstrap values >70% were generated by 1,000 bootstrap replicates using Bayesian inference. Viruses are labeled with different colors depending on the host (red: chicken, orange: environment, blue–green: duck, dark blue: pigeon, dark green: human, purple: wild bird, brown: goose, yellow: mink, light purple: cat). The red circles and font show the six H9N2 AIV strains isolated in this study, and the green font shows human influenza (H9N2). The pink background indicates the Eurasian branch and the blue background indicates the North American branch.

With the continuous evolution of AIV, it gradually evolved into two major branches, the North American and Eurasian branches. The six virus strains of viruses studied are in the Eurasian lineage. The homology of genes within each of the six AIV strains was HA: 91.7%−99.8%, NA: 94.6%−99.6%, M: 94.5%−99.8%, NS: 96.3%−99.4%, NP: 96.7%−99.5%, PA: 94.1%−99.0%, PB1: 93.2%−99.8%, and PB2: 94.9%−97.6%. The evolutionary tree of the HA gene showed two major lineages, Eurasian and North American, and all six virus strains in this research were located in the Eurasian branch. Except for that of the ML3 strain, which formed a separate branch, the HA genes of the other five viruses were clustered with human AIV. The HA gene of the ZJ95 strain was 97.3% homologous to that of the human strain (A/Jiangxi/00346/2021 (H9N2)), that of the DF10 strain was 98.3% homologous to that of A/Guangdong/20SF15010/2020 (H9N2), that of the DF4 strain was 96.7% homologous to that of A/Guangxi/NN10.19T-NGS/2018 (H9N2), that of the ZJ92 strain was 99.3% homologous to that of A/Guizhou/13392/2020 (H9N2), and that of the ZJ81 strain was 98.5% homologous to that of A/Jiangsu/602/2021 (H9N2) (Figures 2 and 3). Similarly, in the NA evolutionary tree of H9N2 AIV, there were two major lineages, and all six virus strains were on the Eurasian branch. In contrast to the ML3 strain, the DF4, ZJ81, ZJ95, DF10, and ZJ92 strains clustered with human influenza virus.

**Figure 4:**
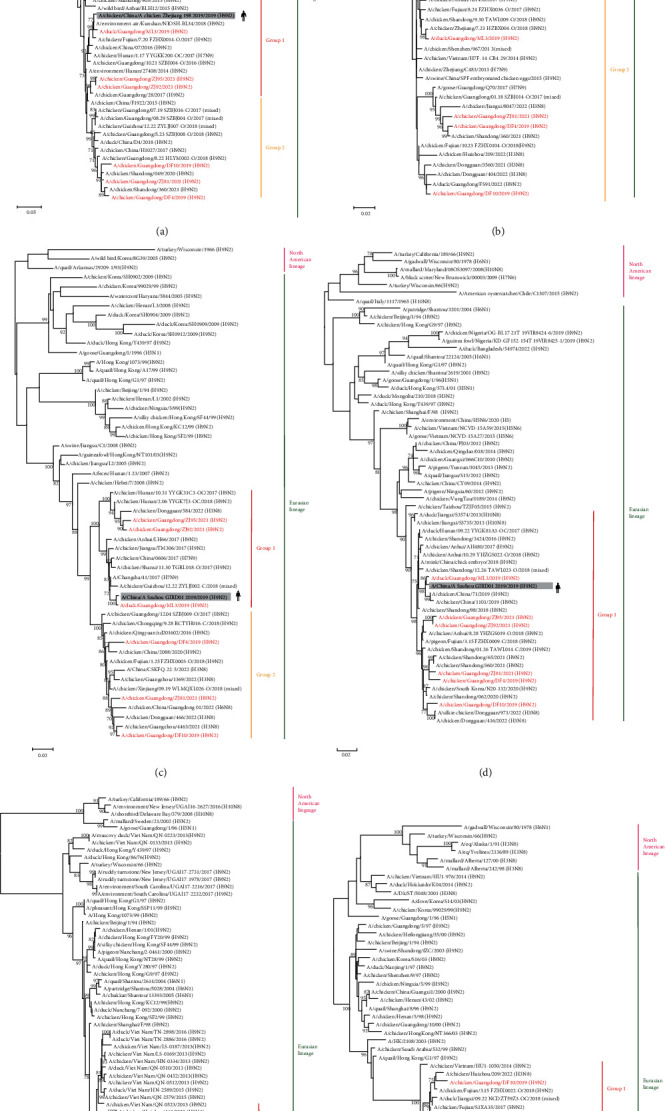
Maximum likelihood-based phylogenetic analysis of the inner genes of H9N2-subtype AIVs isolated from 2019 to 2021: (a) PB2 gene, (b) PB1 gene, (c) PA gene, (d) NP gene, (e) NS gene, and (f) M gene. H9N2 viruses isolated in this study are shown in red. The sequences inside the gray box are from human-infecting AIVs, and those inside the yellow box are from mink-infecting AIVs.

According to the phylogenetic tree, the PB2 genes of the six virus strains were categorized into two groups, with ML3, ZJ92, and ZJ95 being in the same group of PB2 genes as AIV of human origin (A/chicken/China/A chicken Zhejiang 198 2019/2019 (H9N2)), with more than 95% homology (Figure 4). The PA genes of the six H9N2 AIV strains were categorized into two groups. Among them, the PA gene of ML3 is located in the same group as that of the human-origin AIV (A/China/A Suzhou GIRD01 2019/2019 (H9N2)), with 99.3% homology (Figure 4). The NP genes of the six viral strains formed a group with more than 96% homology to the NP genes of AIV of human origin (A/China/A Suzhou GIRD01 2019/2019 (H9N2)). Therefore, the ML3 strain was a chicken–human recombinant virus. The M genes of the six virus strains of viruses in this research belonged to three different groups, of which ZJ81 and ML3 were in the same group. The M genes of two human AIVs, A/Changsha/26/2017 (H7N9) and A/Beijing/1/2017 (H9N2), and A/mink/China/chick embryo/2018 (H9N2) mink AI, belong to the same group as the M gene of ZJ81 virus (Figure 4). The M gene of ZJ81 from the NCBI database has the highest homology of 99.7% with that of A/Beijing/1/2017 (H9N2) (Table 1). Thus, ZJ81 is a chicken–mink–human recombinant virus.

**Figure 5:**
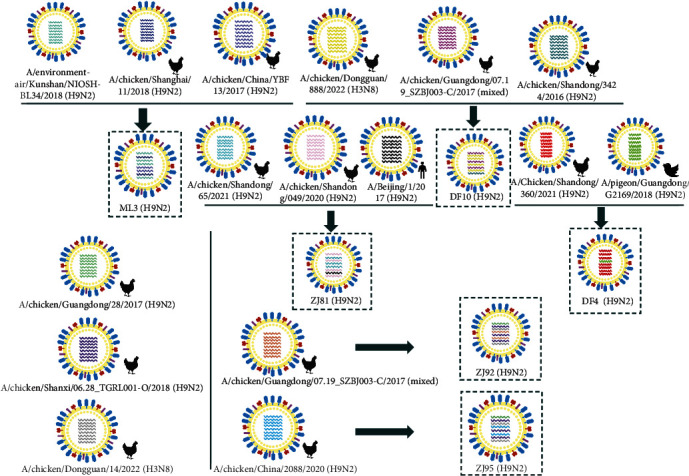
The eight single-stranded RNA fragments expressing the eight genes, namely PB2, PB1, PA, HA, NP, NA, M, and NS (shown by different colored curved lines from top to bottom, respectively). The different colored gene fragments within the virus strains indicate the genetic origin of the virus with the different virus strains.

The genomic origins of these six virus strains were analyzed. The PB2, PA, HA, and NS genes of ML3 were derived from the A/environment-air/Kunshan/NIOSH BL34 (H9N2)-like gene pool, the PB1 and M genes were originated from the A/chicken/Shanghai/11/2018 (H9N2)-like gene pool, and the NP and NS genes originated from the A/chicken/China/YBF13/2017 (H9N2)-like gene pool. The PB2, M, NA, NP, NS, PA, and PB1 genes of DF4 were from the A/chicken/Shandong/360/2021 (H9N2)-like gene pool, and the HA gene was from the A/pigeon/Guangdong/G2169/2018 (H9N2)-like gene pool. The PB2, HA, and NA genes of DF10 were from the A/chicken/Guangdong/07.19_SZBJ003-C/2017 (mixed)-like gene pool, the PB1, PA, NP, and M genes were from the A/chicken/Dongguan/888/2022 (H3N8)-like gene pool, and the NS genes were derived from the A/chicken/Shandong/3424/2016 (H9N2)-like gene pool. The PB2, PA, NA, and NS genes of ZJ81 were derived from the A/chicken/Shandong/049/2020(H9N2)-like gene pool, the PB1, HA, and NP genes were derived from the A/chicken/Shandong/65/2021 (H9N2)-like gene pool, and it is worth noting that the M genes were derived from the human-origin A/Beijing/1/2017(H9N2)-like gene pool. ZJ92 and ZJ95 showed high homology; therefore, the PB2 gene of both virus strains was derived from the A/chicken/Guangdong/28/2017(H9N2)-like gene pool, the PB1, NP, and M genes were derived from the A/chicken/Shanxi/06.28_TGRL001-O/2018 (H9N2)-like gene pool, and both the NS and PA genes were derived from the A/chicken/Dongguan/14/2022 (H3N8)-class gene pool (Figure 5).

### 3.3. Analysis of Key Amino Acid Mutation Site

To further understand the potential threat of these six virus strains, molecular markers for surface and internal genes of these six H9N2-subtype AIVs were analyzed (Table 2). All six virus strains of the cleavage site of the HA gene were PSRSSR/GLF, with no contiguous multiple basic amino acids, implying that these H9N2 viruses had low pathogenicity. The six viruses obtained in this research had the HA-Q226L mutation but not the G228S mutation, indicating that the viruses have a preference to bind to human receptors (*α*-2,6-SA) [29]. The ML3 strain showed a T190V mutation in the HA protein. This means that the virus shows increased replication in mice, and the rest of the virus strains do not have this mutation [30]. Additionally, all six virus strains have the D225G mutation in the HA protein, which suggests the ability to increase transmission and replication in pigs [31]. Of note is the presence of a neck deletion in the NA gene of these six virus strains of the virus, suggesting enhanced pathogenicity in mice [4]. Amino acids 119, 151, 152, 224, 276, 292, and 312 of the NA protein have been associated with protein activity and resistance to the antiviral drugs oseltamivir and zanamivir, but the above mutations were not observed in this study [32].

Amino acid mutations at position E627K and D701N in the PB2 protein imply enhanced viral polymerase activity and viral replication in mammalian cells [33]. None of the six H9N2 viruses obtained here had these mutations. The mutation of aspartic acid for serine at site 66 in the PB1-F2 protein has been associated with raised viral pathogenesis [34], and it is noteworthy that this mutation was present only in ML3. Six virus strains of H9N2 AIVs had a K356R mutation in the PA protein, which increases mammalian replication and pathogenicity, and it has been shown that the presence of this mutation, along with the PB2-E627K mutation, may enable avian H9N2 viruses to infect humans [35]. The PB1 of the six virus strains in this research was mutated at position 622 (D622G), which enhances the toxicity of H5N1 AIV in mice [36]. The NS1 protein had a serine mutation at position 42, and the M1 protein had an Aspartate mutation at position 30 and an Alanine mutation at position 215, implying that the viruses were more virulent in mice. The M2-S31N indicated that these six virus strains had enhanced drug resistance to amantadine [20]. The NS1/NS2 proteins have a mutation in N2D, suggesting increased virulence and IFN-B antagonism [37] (Table 2).

### 3.4. Selection Pressure Analysis

**Figure 6:**
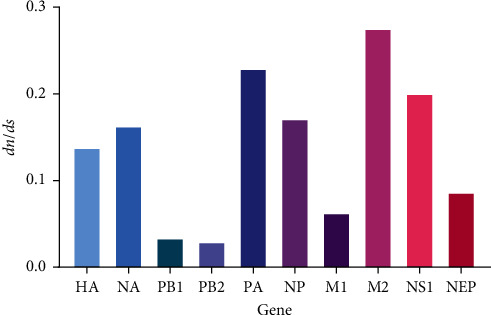
Comparative analysis of the *dN*/*dS* ratio for each gene segment of H9N2 based on the completed alignments.

Selection pressure is one of the main driving forces of viral evolution. Selection pressure acts on a virus through the host immune system when the virus infects host cells, and the virus responds to the pressure through gene mutation or gene recombination mechanisms. Thus, it changes its adaptability and invasion. The evolutionary dynamics of the H9N2 virus were analyzed by calculating the synonymous (*dS*) and nonsynonymous (*dN*) substitutions of its eight gene segments, showing that all segments had *dN*/*dS* < 1, implying that the H9N2 virus experienced negative selection pressure. As the dN/dS ratio approached 1, adaptive evolution occurred. Compared with other gene segments, the PA, M2, and NS1 genes experienced stronger selection pressure, and PB1 and PB2 experienced the weakest selection pressure. It was also found that the *dN*/*dS* of the M2 gene > *dN*/*dS* of the M1 gene and the dN/dS of the NS1 gene > *dN*/*dS* of the NEP gene, which suggested that M2 and NS1 underwent tachytely, in contrast to the M1 gene and NEP gene, respectively (Table 3 and Figure 6).

### 3.5. The HA Protein Structure of H9N2 AIV

**Figure 7:**
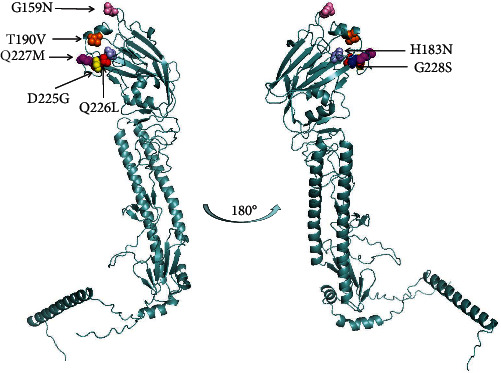
Localization of amino acids related to the antigenicity of H9N2 influenza virus on a 3D map of A/duck/Guangdong/ML3/2019 (H9N2).

The protein structure of the ML3 AIV was constructed, and the positions of the key amino acids are marked with different colors in the figure; they are basically located in the head of the HA protein (Figure 7).

## 4. Discussion

Although H9N2 AIV has low pathogenicity, due to its wide range of hosts, it is capable of breaking through the host barrier and infecting humans and other mammals through genetic mutation and recombination. Poultry infected with H9N2 AIV, especially poultry in live poultry markets, have been shown to be incubators for novel human influenza viruses and are capable of facilitating the spread and evolution of novel human influenza [38]. Therefore, epidemiologic investigation of AIV in live poultry markets is necessary. The worldwide AIV-positive detection rate continued to decline from 2019 to July 2023, especially from January to July 2023, when only five H9N2 AIVs were detected. This may be due to the government's stricter management of live poultry markets, with measures such as regular closures and daily cleaning and disinfection, which have reduced the spread of AIV [23].

Phylogenetic analysis showed that two virus strains, ZJ81 and ML3, were differentially recombined with human influenza viruses. The M gene of the two viruses had the highest homology to that of the human influenza virus. Therefore, ZJ81 is a chicken–human–mink recombinant virus, and ML3 is a chicken–human recombinant virus. In October 2018, an H9N2 AIV infection was detected among chicken, cat, and human populations at a backyard in Guangxi. A human-infecting AIV strain, A/Guangxi/NN10.19T-NGS/2018 (H9N2), and a cat-infecting AIV strain, A/cat/Guangxi/NN10.19H-NGS/2018 (H9N2), from this study were on the same subbranch as the DF4 strain isolated in this study, with homologies of 96.7% and 96.8%, and the homology with ZJ92 and ZJ81 was approximately 95%. This suggests that H9N2 AIV may be transmitted to humans or cats through chickens, increasing the risk of H9N2 AIV infection in humans, and shows that proactive prevention and control measures are warranted [39].

**Figure 8:**
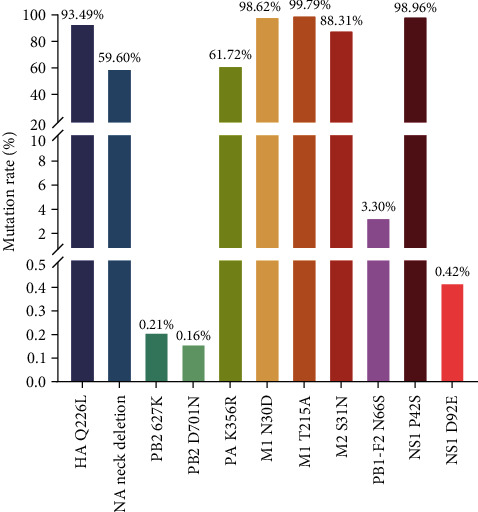
The mutation frequencies of important amino acid sites of H9N2 viruses in the last 5 years from 2017 to 2022 were counted.

Molecular characterization showed that the HA protein of six virus strains had Q226L mutation, implying that the H9N2 strain is shifting from a predilection for avian to human receptors [29]. These six virus strains have three amino acid deletions in their NA protein stems. It has been indicated that NA protein stalk deletion of H9N2 AIV extends the host range, enhances infection of mice, and leads to the generation of mutations in amino acid 627 of the PB2 protein [40]. No amino acids associated with tolerance to anti-influenza drugs such as oseltamivir were found in the NA protein, showing that anti-influenza drugs are still effective against these six virus strains of H9N2 AIV [32]. Statistical analysis of eight gene segments with key amino acid site mutations in H9N2-subtype-AIVs worldwide from January 2017 to July 2023. Mutations at the Q226L site are common in the H9N2 AIV HA protein, suggesting that avian-origin H9N2 viruses generally can bind the human receptor, increasing the public health risk of human infections. Moreover, the M1-N30D and M1-T215A mutations, the M2-S31N mutation, and the NS1-P43S mutation are prevalent in H9N2 AIV, with a mutation rate of more than 88% (Figure 8). All these mutations increase viral pathogenicity in mice, suggesting a potential risk of H9N2 virus infection in humans.

**Figure 9:**
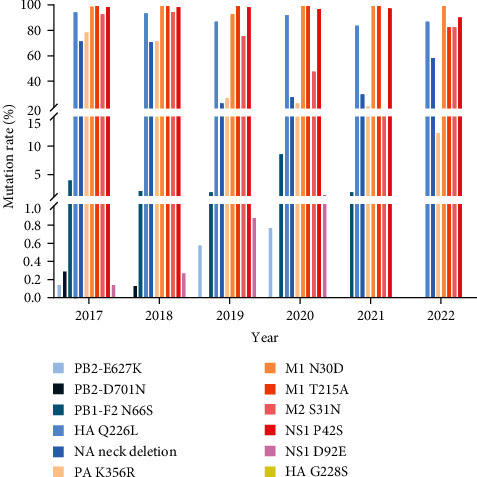
The NA neck deletions of H9N2 sequences and the mutation frequencies of the amino acids HA Q226L and G228S, PB2 E627K, and D701N, PA K356R, M1 N30D, and T215A, M2 S31N, PB1-F2 N66S, NS1 P42S, and D92E were counted for the years 2017–2022.

To further analyze the mutation rules of key amino acid sites, the key amino acid mutations in 2017, 2018, 2019, 2020, 2021, and 2022 were counted, and it was found that the PB1-F2 protein of H9N2 AIV had a lower mutation rate of N66S in each year, except for 2020, which is associated with viral pathogenicity [34]. This mutation was present only in ML3, a chicken–human recombinant virus, among the viruses sequenced in this study. Notably, the rate of NA protein neck deletions and the mutation rate of PA protein K356R have decreased since 2019 (Figure 9), which may be associated with a decrease in the detection rate of AIV after 2019. Therefore, the analysis of mutation trends at key amino acid sites can support early warnings regarding H9N2 AIV.

H9N2 AIV can infect a wide range of hosts, with different evolutionary rates among species. Proteins are considered positively selected when *dN* is greater than *dS*, and *dN*/*dS* < 1 indicates a more conserved protein sequence [41]. A higher *dN*/*dS* signifies the accumulation of adaptive mutations in new hosts [42]. The selection pressure on M2 in this study was greater than that on M1. This means that the positive selection site for M2 is larger than that for M1. It has been suggested that most of the positive selection sites for M2 are located in the extracellular domains and that antibodies recognize the extracellular domains and acquire protective immunity, so the host's immune response may result in higher selective pressure for M2 than for M1 [43]. Selection pressure was greater for NS1 than for NEP, probably because positive selection sites were mainly concentrated on NS1. It has been reported to confirm that the positive selective sites of H1N1 subtype AIV are mostly in the NS1 region, except for the equine lineage [44]. In China, prophylactic vaccination is the main method of controlling the H9N2 virus, but AIV is currently susceptible to antigenic drift due to the rapid evolution of AIV and long-term use of vaccines under immune pressure. It has been shown that some antigen-associated amino acid substitutions, which could cause antigenic drift, challenge vaccine efficacy, and the emergence of a new immune-evasion mutant of H9N2 AIV was reported in southern China in 2020 [45, 46]. Continuous surveillance of H9N2 AIV is, therefore, necessary to aid in the selection of more effective vaccine strains.

## 5. Conclusion

The continued prevalence of AIV of the H9N2 subtype in poultry not only seriously jeopardizes the live poultry markets but also threat to public health security. Furthermore, complex viral recombination may pose a threat to poultry and humans. This study further confirms the prevalence of H9N2-subtype AIV strains worldwide and thus further emphasizes the requirement for the importance of real-time monitoring and control of H9N2 AIV transmission and its importance in poultry farming and human health.

## Figures and Tables

**Table 1:** Six strains of H9N2 AIV with the highest homology in NCBI.

	ZJ81	ZJ92	ZJ95	DF4	DF10	ML3
HA	A/chicken/Shandong/099/2020 (H9N2)	A/chicken/Shandong/360/2021 (H9N2)	A/chicken/Shandong/099/2020 (H9N2)	A/pigeon/Guangdong/G2169/2018 (H9N2)	A/chicken/Jiangxi/X2368/2018 (H9N2)	A/chicken/Jiangsu/J2245/2018(H9N2)

NA	A/chicken/Shandong/049/2020 (H9N2)	A/chicken/Guangdong/08.29_SZBJ025-O/2017 (mixed)	A/chicken/Shandong/049/2020 (H9N2)	A/chicken/Shandong/303/2021 (H9N2)	A/chicken/China/N05/2022 (H9N2)	A/chicken/nantong/02/2017(H9N2)

M	A/Beijing/1/2017 (H9N2)	A/chicken/Shandong/049/2020 (H9N2)	A/chicken/Shandong/049/2020 (H9N2)	A/chicken/Shandong/049/2020 (H9N2)	A/chicken/Fujian/3.15_FZHX0029-O/2018 (H9N2)	A/chicken/Zhejiang/7.23_HZBX014-O/2018(H9N2)

NP	A/chicken/Yunnan/11.22_DQWGH007-O/2018 (mixed)	A/chicken/Fujian/3.15_FZHX0025-O/2018 (H9N2)	A/pigeon/Fujian/3.15_FZHX0009-C/2018 (H9N2)	A/chicken/Fujian/3.15_FZHX0025-O/2018 (H9N2)	A/chicken/Fujian/3.15_FZHX0025-O/2018 (H9N2)	A/chicken/China/71/2019(H9N2)

NS	A/chicken/Vietnam/HU9-506/2018 (H9N2)	A/chicken/Jiangxi/10541/2022 (H3N8)	A/chicken/Guangdong/SD027/2017 (H7N9)	A/chicken/Jiangxi/6959/2022 (H3N8)	A/chicken/Shandong/3424/2016 (H9N2)	A/chicken/China/1104/2019(H9N2)

PA	A/chicken/Guangzhou/17/2019 (H9N2)	A/chicken/Hunan/12.27_YYGK13G3-OC/2017 (H9N2)	A/chicken/Hunan/12.27_YYGK13G3-OC/2017 (H9N2)	A/chicken/Guangdong/1.25_SZBJ014-O/2018 (H9N2)	A/chicken/Guangzhou/4463/2021 (H3N8)	A/China/A_Szuhou_GIRD01_2019/2019(H9N2)

PB1	A/chicken/Shandong/303/2021 (H9N2)	A/chicken/Shanxi/06.28_TGRL001-O/2018 (H9N2)	A/chicken/Shanxi/6.28_TGRL011-O/2018 (H9N2)	A/chicken/Shandong/65/2021 (H9N2)	A/chicken/Guangdong/1.25_SZBJ013-O/2018 (H9N2)	A/environment/Jiangxi/2.21_NCDZT26K2-E/2017(H9N2)

PB2	A/chicken/Guangdong/1.25_SZBJ013-O/2018 (H9N2)	A/chicken/Guangdong/28/2017 (H9N2)	A/chicken/Guangdong/28/2017 (H9N2)	A/chicken/Shandong/360/2021 (H9N2)	A/chicken/Guangdong/1.25_SZBJ013-O/2018 (H9N2)	A/chicken/Fujian/9.25_FZHX0036-C/2017(H9N2)

**Table 2:** Molecular labeling of viral gene segments.

Protein/strain		A/chicken/Guangdong/ZJ81/2021	A/chicken/Guangdong/ZJ95/2021	A/duck/Guangdong/ML3/2019	A/chicken/Guangdong/DF4/2019	A/chicken/Guangdong/ZJ92/2021	A/chicken/Guangdong/DF10/2019
HA	Cleavage site	PSRSSR/GLF	PSRSSR/GLF	PSRSSR/GLF	PSRSSR/GLF	PSRSSR/GLF	PSRSSR/GLF
	Q226L	L	L	L	L	L	L
	G228S	-—	—	—	—	—	—
	H183N	N	N	N	N	N	N
	T190V	—	—	V	—	—	—
	Q227M	M	M	M	M	M	M
	G159N	—	—	N	—	—	—
	D225G	G	G	G	G	G	G

NA	Neck deletion	TEI	TEI	TEI	TEI	TEI	TEI
	E119	—	—	—	—	—	—
	D151	—	—	—	—	—	—
	R152	—	—	—	—	—	—
	R224	—	—	—	—	—	—
	E276	—	—	—	—	—	—
	R292	—	—	—	—	—	—
	I312	—	—	—	—	—	—

PB2	E627K	—	—	—	—	—	—
	D701N	—	—	—	—	—	—
	G309D	D	D	D	D	D	D
	T339K	K	K	K	K	K	K
	A588V	V	V	V	V	V	V
	L89V	V	V	V	V	V	V
	R477G	G	G	G	G	G	G
	I465V	V	V	V	V	V	V

PB1	D622G	G	G	G	G	G	G
	I368V	V	V	V	V	V	V

PB1-F2	N66S	—	—	S	—	—	—

M1	N30D	D	D	D	D	D	D
	T215A	A	A	A	A	A	A

M2	S31N	N	N	N	N	N	N

NS1	P42S	S	S	S	S	S	S
	D92E	—	—	—	—	—	—

NEP	N2D	D	D	D	D	D	D

PA	S37A	—	—	—	—	—	—
	N383D	D	D	D	D	D	D
	K356R	R	R	R	R	R	R

*HA residue positions are presented with H3 number, NA residue positions are presented with N2 number*.

**Table 3:** Results of the *dn*/*ds* calculations.

Mean *dn*/*ds*	*dn*	*ds*	*dn*/*ds*
HA	0.04066	0.29433	0.13814
NA	0.05527	0.33989	0.16261
PB1	0.01229	0.36571	0.03361
PB2	0.01065	0.36470	0.02920
PA	0.01249	0.33364	0.22927
NP	0.01162	0.33032	0.17097
M1	0.01438	0.22980	0.06258
M2	0.03461	0.12572	0.27529
NS1	0.04548	0.22706	0.20030
NEP	0.02176	0.25260	0.08614

## Data Availability

The data used to support the findings of this study are included within the article.
